# A 5000 Fps, 4 Megapixel, Radiation-Tolerant, Wafer-Scale CMOS Image Sensor for the Direct Detection of Electrons and Photons †

**DOI:** 10.3390/s26020370

**Published:** 2026-01-06

**Authors:** Andrew Scott, Claus Bauzà, Adrià Bofill-Petit, Albert Font, Mireia Gargallo, Robert Gifreu, Kamran Latif, Armand Mollà Garcia, Michele Sannino, Renato Turchetta

**Affiliations:** IMASENIC S.L., C. Torrassa 77, Sant Adrià de Besòs, 08930 Barcelona, Spain; claus.bauza@imasenic.com (C.B.); adria.bofill@imasenic.com (A.B.-P.); albert.font@imasenic.com (A.F.); mireia.gargallo@imasenic.com (M.G.); robert.gifreu@imasenic.com (R.G.); kamran.latif@imasenic.com (K.L.); armand.molla@imasenic.com (A.M.G.); michele.sannino@imasenic.com (M.S.); renato.turchetta@imasenic.com (R.T.)

**Keywords:** wafer scale, stitching, CMOS image sensor, high frame rate, high speed, rad-tolerant

## Abstract

We present the design and characterisation of a 4.2-megapixel, wafer-scale CMOS image sensor, achieving over 5000 frames per second at full resolution. The sensor has a pixel pitch of 58 µm square pixels, thus being as large as a full 200 mm wafer. The sensor is read out on two sides and features column-parallel programmable gain amplifiers (PGAs) as well as analogue-to-digital converters (ADCs). The array has 2052 columns and 2064 rows; 12 rows are read in parallel, so that the total number of ADCs is 24,624. The data is transmitted through 216 sub-LVDS lanes running at 1 Gbps in double data rate (DDR). Besides the row and column control, the sensor generates the necessary voltages and currents on a chip. The programming is performed through a serial-to-parallel interface (SPI). The sensor was optimised for direct detection of electrons, but it can also detect photons. Thus, it could be a good candidate for applications where high speed is needed, such as wavefront sensing.

## 1. Introduction

**Figure 1:**
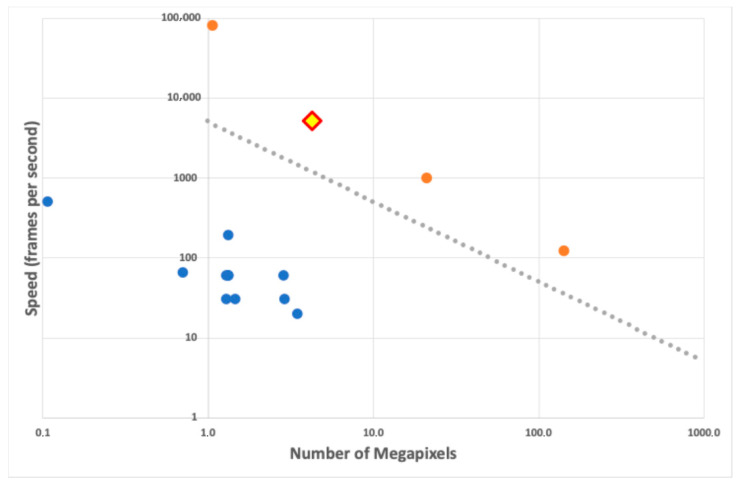
Speed (given in megapixels per second) as a function of array size (in megapixels) for existing CISs. The blue dots correspond to wafer-scale sensors; the orange dots correspond to high-speed CISs; the grey dotted line represents the line of equal pixel rate, which, in this case, is 5 Gpixel/sec. It is clear that wafer-scale sensors lie well below this line. The sensor presented in this paper is shown in the plot as the yellow diamond with a red border; it lies well above the grey line, in an area until now occupied only by conventional high-speed CISs.

Wafer-scale CMOS image sensors (CISs) have been developed for many years, mainly for X-ray detection [1,2,3,4]. Despite continuous improvements, their speed tends to be limited to a few hundred megapixels per second, while conventional high-speed sensors, with a smaller size, normally limited to a reticle, can achieve pixel rates of a few tens of gigapixels per second [5,6,7]. Figure 1 shows data from existing wafer-scale sensors, as well as from some high-speed CISs. The grey line is a line of constant pixel rate at 5 Gpixel/second. There is a significant gap between the two above-mentioned categories. The figure also shows the point corresponding to the sensor presented here; working with an 8-bit resolution, this point is well aligned with the high-speed category and so will the corresponding points for higher bit depths of 9 or 10 bits. Our sensor thus clearly breaches the gap in speed between conventional high-speed CISs and wafer-scale ones. It was designed for the direct detection of electrons, but it can also detect light, as its characterisation results show. It can also be used for other applications where speed and a large area are required, such as high-speed X-ray detection coupled to a suitable scintillator, or for high-speed visible-light imaging.

The lead application is Cryo-Electron Microscopy (Cryo-EM), where transmission electron microscopes are used. The importance of detectors in Cryo-EM was already recognised in 2014 [8]. In 2017, in his Nobel Prize award lecture, Prof. R. Henderson talked about the importance of detector development in improving the performance of Cryo-EM. Today, Cryo-EM is about to become the leading tool for the reconstruction of complex biological structures, as shown in the statistics released by the Protein Data Bank [9]. Cryo-EM also played an important role in determining the structure of the COVID-19 virus, as shown in [10,11]. Until now, most high-resolution cryo-EM microscopes have worked at a relatively high voltage of 300 kV [12]. In order to reduce the cost of these machines, it was proposed to reduce the voltage applied to the electrons; hence, their energy was reduced to 100 keV, and it was demonstrated that, despite the reduction in energy, the image quality remains high [13]. More recently, more structures have been determined, proving that the resolution can be maintained whilst reducing the energy [14,15].

Reducing the energy of the electrons requires new sensors because of the physics of the interaction of the electrons with the sensor [16,17]. Three routes have been followed on the way to developing such new sensors: adapting existing CIS, using hybrid pixel sensors, and developing new CIS, optimised for this reduced energy.

In the first set of sensors fall the Gatan’s Alpine [14] or Thermo Fisher’s Falcon C [15]. Dectris, the company leader in hybrid pixel sensors, have developed a product for Cryo-EM, called Singla [18]. In the last category of developing an optimised CIS, one can find the sensor developed by a research consortium in the UK, formed by the company Quantum Detectors, together with the Science and Technology Facilities Council (STFC) and the Rosalind Franklin Institute (RFI) [19]. In this paper, we present the development of a new CIS optimised for direct detection of 100 keV electrons. Results from a prototype were already presented in [20], and here we present the design and characterisation of the final sensor, a 4 Mpixel, wafer-scale sensor designed and manufactured in a 180 nm CIS process.

Lowering the electron energy from 300 keV to 100 keV increases energy loss (see Figure 1 in [20]) and scattering, affecting charge distribution and sensor response, resulting in the requirement for larger pixels [16,17]. The prototype had 60 µm square pixels, and in the final product, the size was slightly reduced to 58 µm in order to accommodate a greater number of pixels within a single 200 mm wafer. Consequently, the final sensor has a format of 2052 × 2064 or 4.2 million pixels, with a focal plane size of 119.0 mm × 119.7 mm.

Regardless of electron energy, biological samples in Cryo-EM are subject to radiation damage from the electron beam. To mitigate this, data must be collected as quickly as possible. With the intention of achieving the highest spatial resolution, the charge generated by a single 100 keV electron needs to be carefully analysed. This requires every frame to have a low density of electrons in an effort to avoid pile-up. These two requirements, radiation hardness of the sample and single-electron detection, demand high frame rates. The target specification is to achieve 5000 frames per second. Combined with the sensor format, this gives a pixel rate in excess of 20 Gpixels per second. This requires the sensor to be fully digital, with a high-speed digital interface for data and a serial interface for the control of the chip. The design will be described in detail in Section 2. Experimental results will be presented in Section 3 before closing the paper with the conclusions in Section 4.

## 2. The Design

**Figure 2:**
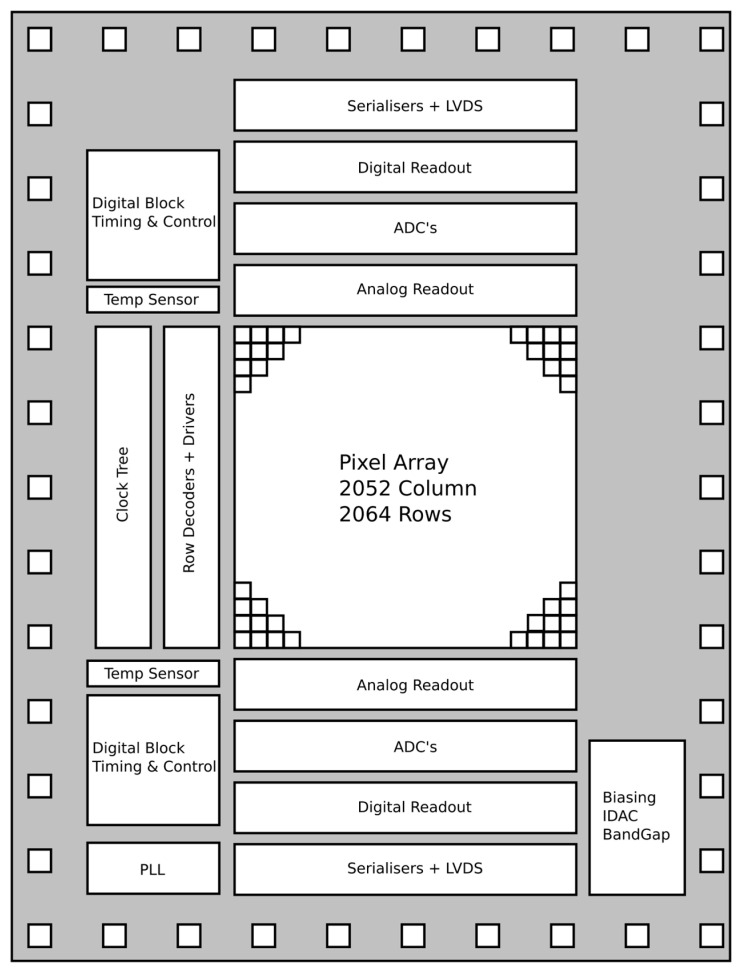
Floorplan of the sensor. It features a pixel array with readout on the top and bottom sides. The readout includes an analogue readout featuring PGAs, incremental sigma-delta ADCs with a resolution up to 10 bits, and digital readout via subLVDS. The chip generates on-chip references with the biasing block. Digital control is provided via the two digital blocks on the top and bottom sides, with the PLL providing the clock for the sensor. Finally, the chip features two temperature sensors, one on the top and one on the bottom side.

The floorplan of the sensor is shown in Figure 2. Together with the pixel array, it features all the blocks typical of a camera-on-a-chip CIS. The readout can be found at the top and bottom of the pixel array, and it includes programmable gain amplifiers (PGAs), sample-and-holds, and incremental sigma-delta analogue-to-digital converters (ADCs) with an adjustable resolution between 4 and 10 bits. Digitised data are serialised and transmitted to the outside world through subLVDS output pairs. Currents required by the analogue blocks are generated on-chip with current digital-to-analogue converters (iDACs). If needed, these currents are converted to voltages before being fed to the biasing block. Digital control is provided via the two digital blocks on the top and bottom sides. These blocks also contain the registers storing timing information, as well as the serial-to-parallel interface (SPI) over which the chip receives data and commands from the outside world. The phase-locked loop (PLL) block generates a high-frequency clock from an input lower-frequency clock, typically running at 20 MHz. Finally, the chip features two temperature sensors, one on the top and one on the bottom side.

### 2.1. Stitching Plan and Architecture Decisions

Building on the sensor design requirements outlined in the introduction, we now describe the readout architecture, which was driven by the requirement of a large 2 k × 2 k pixel array and a frame rate of 5000 fps. These specifications impose timing and bandwidth constraints, both at the pixel level and at the data output stage.

The large array size and high frame rate leave very limited time for transferring data from the pixel array and necessitate a high-bandwidth acquisition system. The effective line time, with some rounding, is 100 ns (calculated as 1/5000 fps × 1/2000 rows), while the corresponding raw data rate reaches 180 Gbps (2000 × 2000 × 9 bits × 5000 fps). With only 100 ns available per line, stable pixel output could not be achieved.

To address this limitation, a simultaneous readout of 12 rows was implemented, yielding a more manageable row-group time of 1.156 µs. The readout lines are split at the centre of the array, dividing it into top and bottom halves. Six rows from each half are read together to form the 12-row readout group. Readout proceeds outward from the centre: upwards for the top half and downwards for the bottom half.

**Figure 3:**
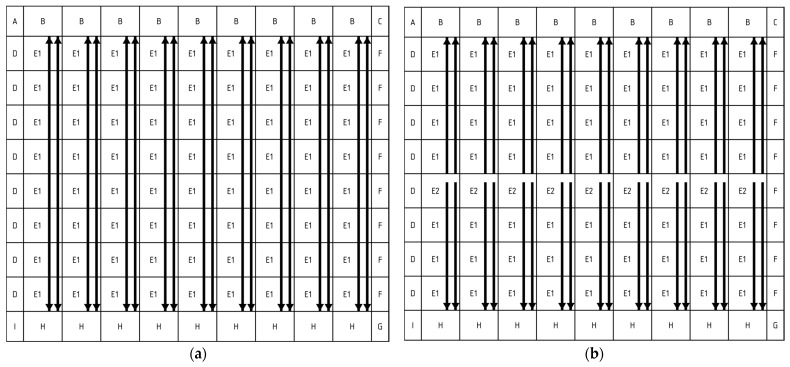
Organisation of the sensor with 9 horizontal and 8 vertical repetitions. The arrows show how the pixels are connected to the readout. (**a**) shows the typical stitching plan where only one block E mask is used, and therefore, the output lines are connected at the top and bottom. (**b**) shows how the output lines are split, with the pixel block E having two versions of the mask E1 and E2, where E2 is used to generate the split between the top and bottom output lines. The stitching pattern shown in (**b**) was only used for one mask; all the others followed the standard pattern shown in (**a**).

In a stitched sensor, all blocks containing pixels are identical, as shown in Figure 3a. With the aim of splitting the readout lines in the middle of the array, all these lines were implemented in the same metal level. Two different masks were fabricated for this metal layer: one applied exclusively to the central row of stitching blocks, referred to as E2, and another applied to all remaining pixels blocks, referred to as E1; (see Figure 3b). Splitting the readout lines at the centre of the array reduces the output line capacitance by half, improving settling time but introducing an additional constraint that the number of vertical pixel block repetitions must be even to guarantee an equal number of pixels on each side of the array.

The number of sub-LVDS lanes was another key design factor. The sub-LVDS transmitter was designed for up to 1.2 Gbps with double data rate (DDR) to give some margins on the required readout speed of 1 Gbps. DDR was chosen to keep the required clock frequency to 500 MHz, which is possible to produce with an on-chip PLL. Under these conditions, a single sub-LVDS lane can support a maximum of $\frac{1.156\mu s\;(row\;group\;time)*1Gbps\;(datarate)}{9b(bits\;per\;pixel)*6\;rows}=21.4$ columns. Based on this limit, fitting the required number of pixels and satisfying the design rules for stitched sensors, we allocated 12 sub-LVDS lanes per stitching block, each serving 19 columns. This results in 228 columns per stitching block and 9 horizontal repetitions, as shown in Figure 3b.

To reduce the design and verification complexity, and consequently the design time, active pixels have only been placed in the central repeating stitching blocks E. As a result, no active pixels are in the non-repeating stitching blocks, B, D, F, and H, at the left and right edges of the sensor, as well as at the top and bottom.

**Figure 4:**
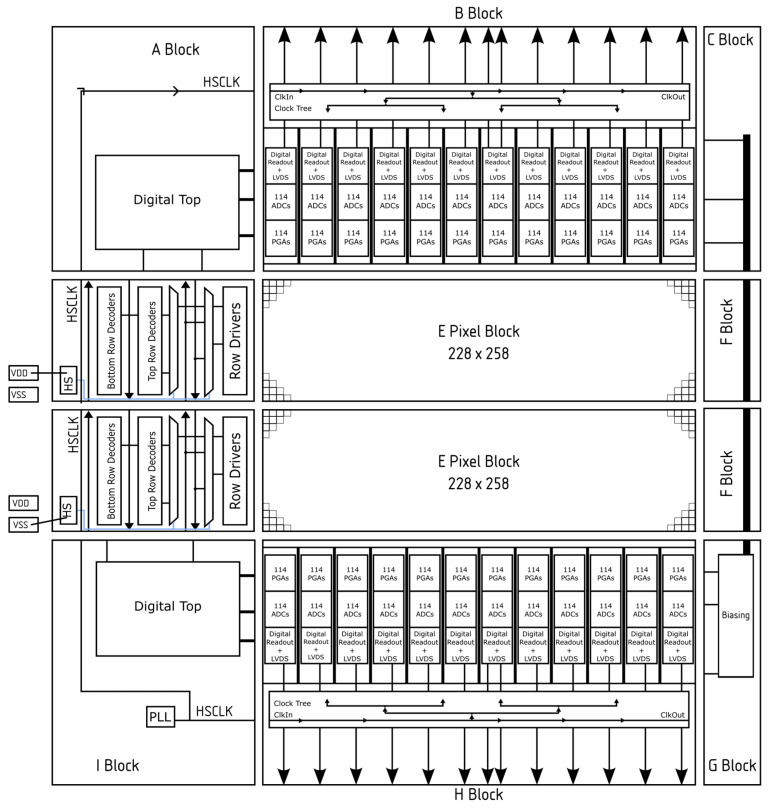
Stitching plan and organisation of the sensor shown as a 1 × 2 sensor, including the stitching block number, which can be combined with Figure 3b and includes how the Half Select (HS) signal is used to select the row decoder to be used, shown in blue. The LVDS lines extend from the LVDS driver to the edge of the block. In total, there are 12 data pairs and one clock pair per H or B block. Arrows also indicate the direction of signals, such as the routing of the clock or the transfer of signals from the two digital tops to the row decoders/drivers.

In total, the sensor integrates 108 sub-LVDS lanes per half, resulting in 216 lanes overall. The High-Speed Clock (HSCLK) is distributed around the sensor. Once within a stitching block, it is sent through a clock tree in order to minimise the skew within the stitching block, as seen in Figure 4. Together with the 12 sub-LVDS data pairs, an extra sub-LVDS pair is added in each stitching block to output the clock for source-synchronous sampling. Source-synchronous data transfer was necessary to maintain timing integrity, given the number of sub-LVDS lines and the distance the output signals needed to travel. This distance is relatively high, due to the sensor being placed within the microscope column and thus within a vacuum. This environment is not suitable for FPGAs to be placed due to the radiation damage and lack of cooling without adding a significant amount to the system cost. Any additional skew within one stitching block will be accounted for within the readout FPGA. Figure 4 illustrates the sub-LVDS lane organisation where the arrows indicate a sub-LVDS lane, with the clock being output in the centre of the stitching block.

The vertical stitching plan was constrained by several factors, including accommodation of 2000 rows, enforcement of an even number of pixel-block repetitions to enable pixel-output splitting, and integration of the readout circuitry at the top and bottom within the available mask height.

The pixel block comprises 258 rows and is replicated eight times in the vertical direction, satisfying the even-repetition requirement while meeting overall array size constraints. This results in a total of 2064 rows, slightly exceeding the target value. The additional capacitance introduced by the extra 64 pixels is negligible relative to the total array capacitance.

A region-of-interest (ROI) readout mode enables acquisition from smaller regions by selecting a reduced number of rows. The ROI is always centred on the sensor, with an equal number of pixels read from the upper and lower halves of the array.

**Figure 5:**
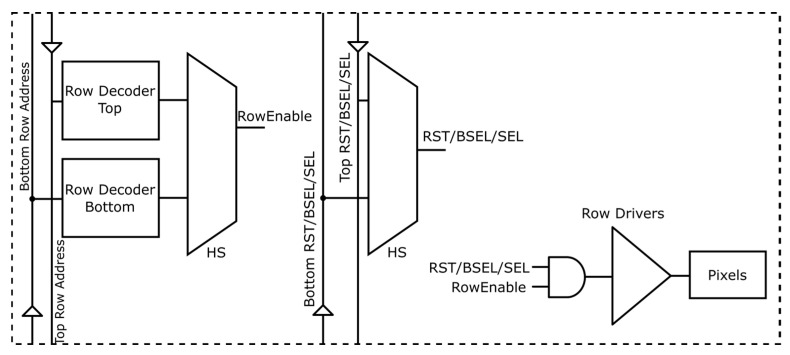
Schematic of the top- and bottom-row decoders showing buffering of pixel timing signals (e.g., row address, RST) distributed from the top or bottom of the sensor, as selected by the HS signal. Signal routing is buffered from top to bottom or from bottom to top, depending on the signal direction; the triangle denotes a buffer.

The resulting stitched array comprises 2064 rows × 2052 columns and incorporates 12 ADCs per column, yielding a total of 24,624 ADCs. As previously mentioned, the pixel array is split into two halves, controlled independently by two identical digital control (digital top) blocks, located at either the top or bottom of the sensor. The routing for the timing signal associated with the readout, such as the signals for the programmable gain amplifiers (PGAs), sample and hold (S/H), and ADCs, can be distributed horizontally in a traditional manner for these signals. For example, the bottom digital control block provides the PGA signals via horizontal routing. The pixel signals are more complicated due to the row decoders taking signals from both the top and bottom digital blocks, and they must choose which signals to use. This selection cannot be physically implemented within the layout, as the stitching block must be identical regardless of its location in the array. To resolve this, two decoders were implemented: one driven from the top digital control and the other from the bottom digital control block, with a multiplexer selecting between them. The selection is taken care of via a pad, named Half Select (HS), from within the stitching block. By bonding the HS pad to either VDD or VSS (see Figure 4 and Figure 5), the decoder and timing signals (e.g., RST, SEL) are multiplexed consistently, ensuring that skew between the top and bottom halves does not affect operation. The two halves of the sensor can be considered independently in terms of timing, thus helping to maximise the frame rate from the sensor.

Although most of the chip is duplicated symmetrically between the top and bottom halves, a few critical blocks are shared. A single PLL is used to provide a common clock, which is routed upward and then distributed symmetrically to both halves. Naturally, since there is only one PLL and it has been placed on one side of the chip, there will be a skew in timing between the top half and the bottom half. This skew was assessed to be only a few cycles of the system clock and thus would not cause any issues since the two sides of the chip work independently. Similarly, the biasing block, V to I converter, and band gap are individually integrated in the bottom-right corner of the sensor (see Figure 2), and the resulting voltages and currents are distributed across the full array.

### 2.2. Pixel

**Figure 6:**
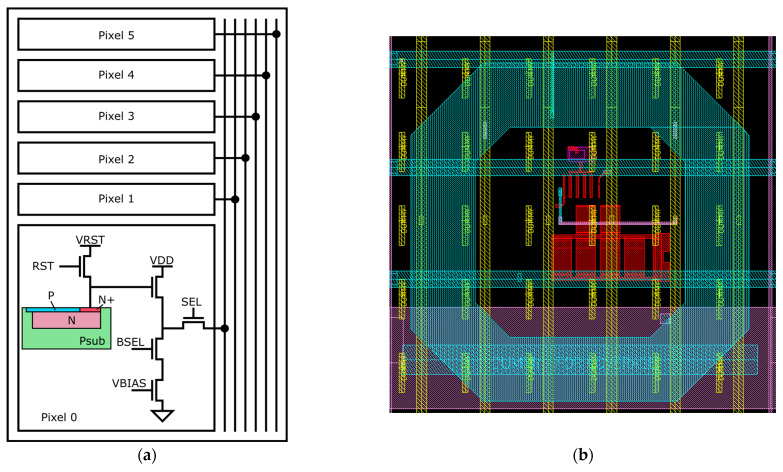
(**a**) Pixel schematic and grouping for one side of the sensor. Six pixels within each group are read out to the same side, with a corresponding group of six pixels read out to the opposite side of the sensor. (**b**) Pixel layout showing the donut diode (cyan), source follower, and biasing transistors located at the centre of the diode (red), as well as output lines in yellow. Control passing across the pixel is in cyan and the distribution of the bias is in pink.

The pixel, whose schematic is shown in Figure 6, has a 3T-like architecture with local biasing, with a Partially Pinned Photodiode (PPPD) [2], a reset, source follower, select, bias select, and bias. The pixel differs from typical 3T pixels not just from having the local biasing, but also due to the reset transistor being a PMOS. A PMOS device was chosen for the reset transistor to achieve faster reset performance. The PPPD allows us to keep the input capacitance low, even for a large diode. The PPPD is formed by a N+ implant in a P- substrate, with a shallow P+ pinning layer covering most of the photodiode, as shown in Figure 6a. To prevent charge loss due to collection by the PMOS n-well, the latter was enclosed within a deep p-well implant [21].

Unlike conventional column-shared designs, the bias circuit is implemented per pixel, which improves the output voltage range and ensures uniformity in the layout when splitting the output line in the middle. 1.8 V transistors are used instead of 3.3 V ones in order to take advantage of the thinner oxide layer, improving radiation hardness with respect to thicker oxide devices [22]. In an electron microscope, it is possible to protect the periphery from direct hits of the electrons; the most challenging radiation resistance requirements lie in the focal plane. In general, the radiation hardness of the sensor has to be good enough so that the sensor can survive several years of usage in a cryoEM. The radiation hardness of the pixel was tested in the prototype and proven to be adequate [13] for this lifetime requirement. Since the design only uses 1.8 V transistors, rather than the 3.3 V typically used in pixel designs within this technology, the output range is reduced. The bias was placed locally to avoid having the current pass through the selected transistor, which helps to maximise the output voltage range of the pixel compared to a traditional design.

The pixels are grouped in sets of six within a row group, with common timing signals shared across the group. The group of six is read from one side of the chip, with an identical and symmetrically placed group of six being read on the other side of the chip. The upper metal layers were used to form supply meshes for both VRST and VDD, while signal lines were carefully routed to maximise yield. For large-area CMOS sensors, since the pixel arrays are typically over 90% of the area of the chip, it is a common requirement to place dummy metals to meet coverage requirements.

### 2.3. Analogue Readout

**Figure 7:**
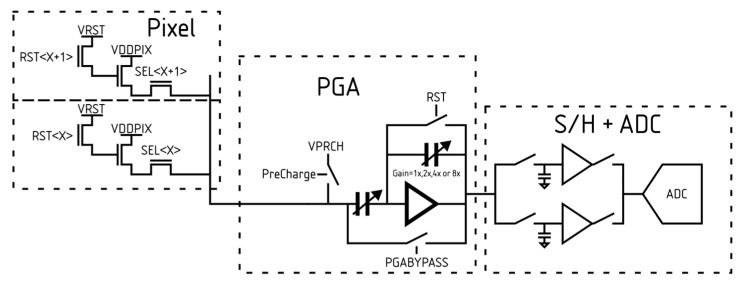
Schematic of signal path including a simplified version of the pixel, PGA, S/H, and ADC structure.

The schematic of the signal path is shown in Figure 7. The pixel outputs are read through column-parallel programmable gain amplifiers (PGAs), with selectable integer gain values from 1 to 8. It is also possible to bypass the PGA and connect the pixel output directly to the sample-and-hold stage. In the case a unity gain is needed, this bypass connection helps maximise the performance by reducing the noise and power. The sample-and-hold employs two interleaved branches operating in turn to maximise readout speed.

**Figure 8:**
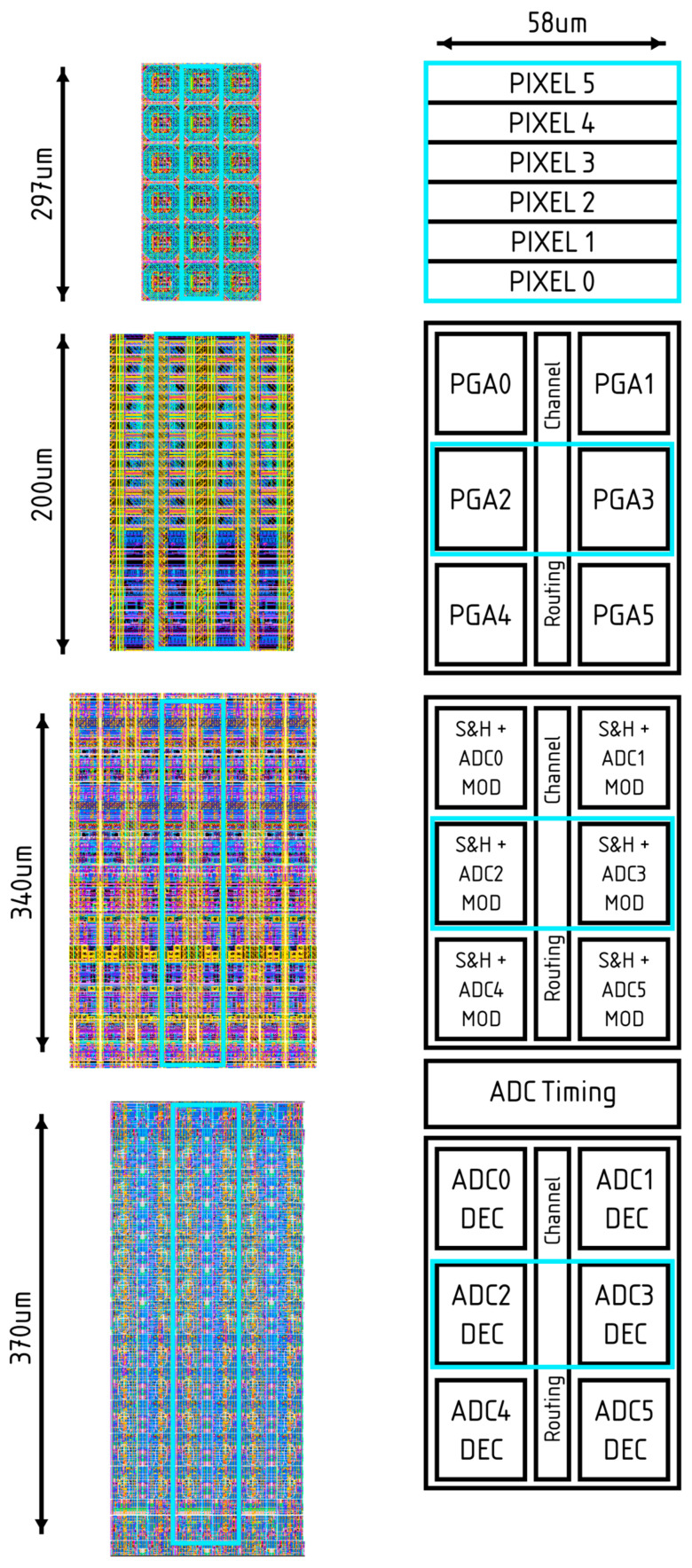
Organisation of the analogue readout for one column, including snippets of the layout. The cyan box highlights the regions shown in the layout and their correspondence with the block diagram.

Within each column, there are 6 analogue readout structures. The column is organised such that there are 3 rows of 2 units, separated by spacing that allows routing channels for signals and power within the array. Figure 8 shows the structure and layout used for each component within a column. This structure has been used to provide greater flexibility in cell sizing, allowing components such as MIM capacitors to be placed without interrupting power-supply routing, while also enabling signals to pass through with shielding to mitigate crosstalk. Managing crosstalk is particularly important when using multiple rows within the readout structure since the neighbouring readout can be several pixels away when referred to the pixel locations.

**Figure 9:**
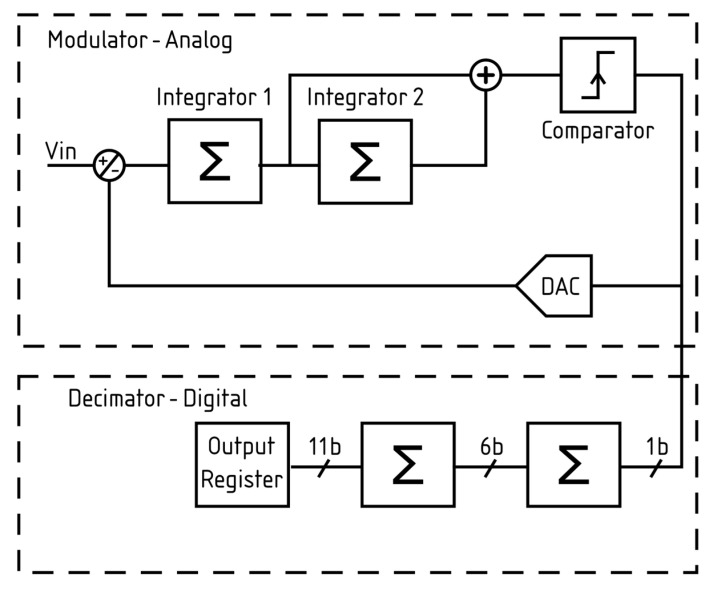
The block diagram of the Incremental sigma-delta ADC.

Each branch is followed by a unity-gain buffer that drives a second-order incremental sigma-delta ADC, whose architecture is shown in Figure 9 and originally described in [22]. This version of the ADC uses the same topology but employs 1.8 V transistors, rather than a mixture of 1.8 and 3.3 V devices, resulting in a different layout. The use of the 1.8 V transistors has allowed the ADC to be run with a higher ADC clock of 35.7 MHz rather than the 25 MHz that was used in the original design. The bit depth of the ADC was reduced to meet the required amount of up to 10 bits. The bit depth can also be programmed to be 4, 6, 8, 9, or 10 bits, thus allowing trade-offs between accuracy and speed. By default, a bit depth of 9 was selected. All the results presented here were obtained with this bit depth.

**Figure 10:**
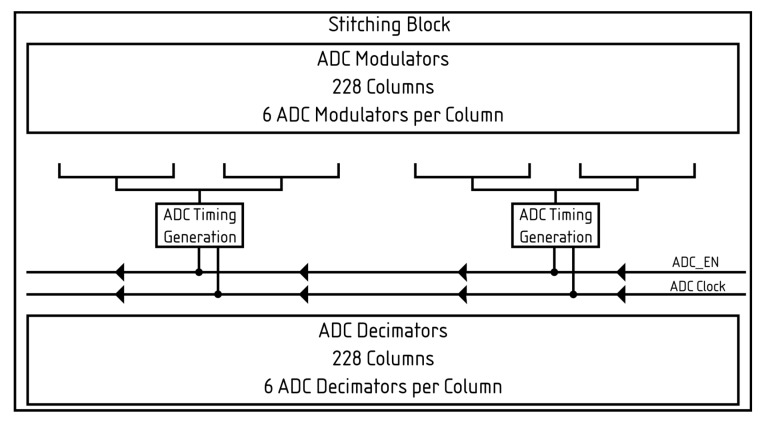
Diagram showing how the ADCs are distributed within the stitching block, including how the ADC timing is generated from the ADC clock and ADC enable signals. These signals are passed across the stitching block into the next stitching block. The black lines with arrows indicate that the signal is buffered as it passes across the stitching block.

The individual layout and distribution of the incremental sigma-delta ADC signals are important, especially on a wafer-scale design. Figure 10 shows how the ADC signals are distributed across the stitching block with two clock generation blocks per stitching block. The digital top produces two signals for the clock generation blocks. The first is the ADC clock, which is used to produce timing signals for the ADC, and the second is the ADC enable. The ADC enable is sampled with the ADC clock and starts producing the required ADC signals. The falling edge of the ADC enable is used to end the conversion and load the output registers. The length of the enable is programmable via a configuration register to adjust the number of cycles the ADC converts, thus controlling the ADC resolution. These signals, output from the clock generation block, are distributed via a clock tree to ensure timing is kept. The placement of the ADC timing generation cell between the two parts of the ADC was performed to minimise the routing required for the timing signals since, some of the signals go to both the modulator and decimator, reducing the length of wire required and thus the power.

The ADC conversion result is stored in an 11-bit register, providing a margin for over-range even at the maximum 10-bit resolution. Since the required ADC resolution is lower than in 14-bit case in [22], the number of ADC cycles needed for the conversion was lowered to 42 cycles for the 9-bit case. Using the maximum ADC clock frequency of 35.7 MHz, we achieved a 9-bit conversion in 1.18 µsec. The bit depth is user-selectable between 4 and 10 bits. When reducing the bit depth, both the ADC resolution and the output data format are adjusted to maximise the framerate. The number of ADC cycles, which controls the accuracy of the conversion used in the ADC, is configured according to the selected bit depth, such that the conversion time decreases for lower bit depths and increases for higher bit depths.

The physical arrangement of PGAs and ADCs is illustrated in Figure 8. Careful layout techniques were required to minimise crosstalk both between adjacent columns and within each column (PGA-to-PGA and ADC-to-ADC). This consideration is particularly important when multiple rows are read simultaneously, as crosstalk can occur between distant rows, thus degrading the overall image quality.

### 2.4. Digital Readout

The digital readout block acquires data from the ADC array, applies column fixed-pattern noise (FPN) subtraction, formats the output according to the selected bit depth, and prepares it for serialisation and transmission via the sub-LVDS lanes. The system supports programmable bit depths of 4, 6, 8, 9, or 10 bits, with the resulting data packed into 16-bit words for all configurations. Each digital readout block serves 19 columns and 6 rows.

**Figure 11:**
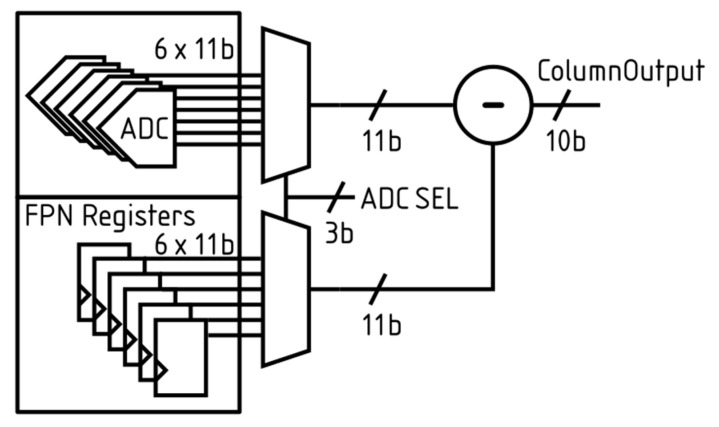
Illustrates the FPN cancellation circuit and highlights how data is output from each column.

The digital readout provides each column with a 3-bit select signal (ADC_SEL in Figure 11) to determine which row (0–5) within the row group from the column should be output. The column then provides a 10-bit value from the selected row. The digital readout then samples the required data from the 10-bit number before incrementing the row selection, ensuring that subsequent samples are given the maximum settling time.

To minimise area overhead, the memory required for data packing is implemented within the ADC array, avoiding duplication in the digital block. The digital block then outputs the pixel data in raster order, e.g., row 0 columns 0–18, followed by row 1 columns 0–18, and so forth.

To maximise the effective number of bits in the output, a column fixed-pattern noise (FPN) correction scheme has been implemented. A dark reference dataset is first captured to determine the ADC offset values. These values are then written back to the sensor via the SPI. Each ADC stores its own offset value locally to the subtractor, as seen in Figure 11, to minimise the distance the data must travel. Once the ADCSEL has been provided, a multiplexer selects the corresponding ADC and FPN register for the subtractor, producing a 10-bit column output with the column FPN compensated. Should raw data from the ADC be required, this can be obtained by keeping the FPN registers in reset. This setting is used when acquiring the dark reference dataset. For area efficiency, a single subtractor is used in each column. Sharing subtractors across multiple columns was not feasible owing to the high frame rate and bit-depth flexibility required. Figure 11 illustrates the implementation of the FPN cancellation circuit and highlights the role of the ADC_SEL signal in selecting rows for readout.

### 2.5. Readout Timing

**Figure 12:**
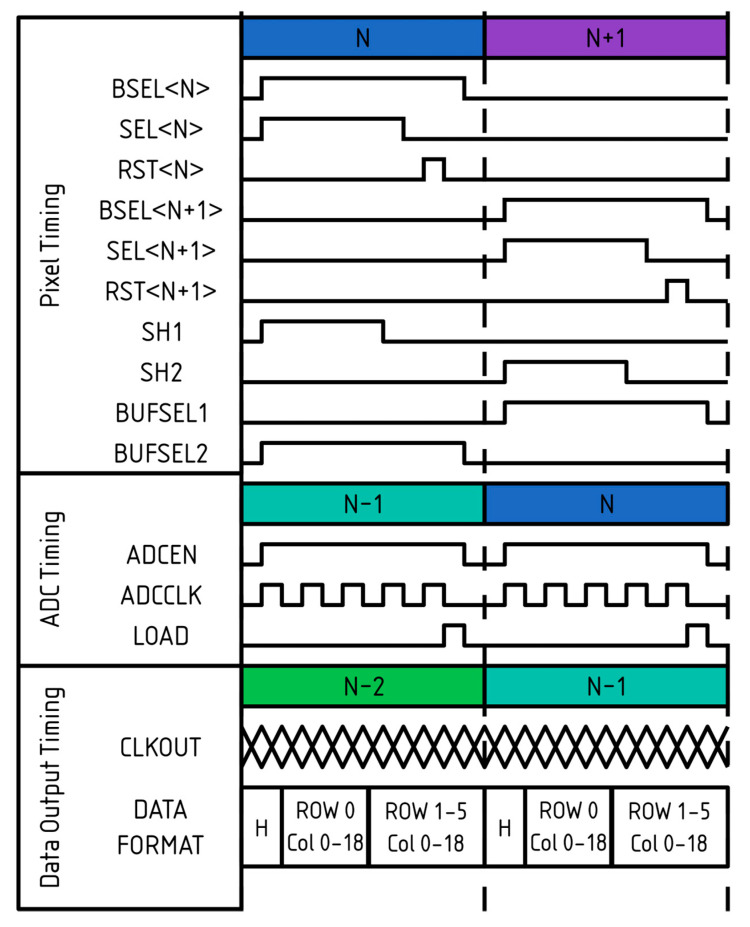
A timing diagram showing the sensor timing used when the PGA is bypassed.

An overview of the timing is shown in Figure 12. The example corresponds to operation with the PGA bypassed; therefore, no PGA control signals are present. The pipelining scheme is highlighted, illustrating how row groups are managed as they progress through the readout stages, including the ping-pong sample-and-hold stage.

Pixel timing is shown for two row groups, where BSEL, SEL, and RST are applied to all six pixels within each group. Once the pixels have been read and sampled by either SH1 or SH2, the pixel is reset in preparation for the next frame. The sampled values are then held for ADC conversion before being output, as illustrated in the data output timing section.

The data format is also shown for a sub-LVDS output. As described previously, each sub-LVDS channel serves 6 rows and 19 columns. Data are transmitted sequentially, row by row, and this ordering is maintained independently of the selected bit depth.

## 3. Results

**Figure 13:**
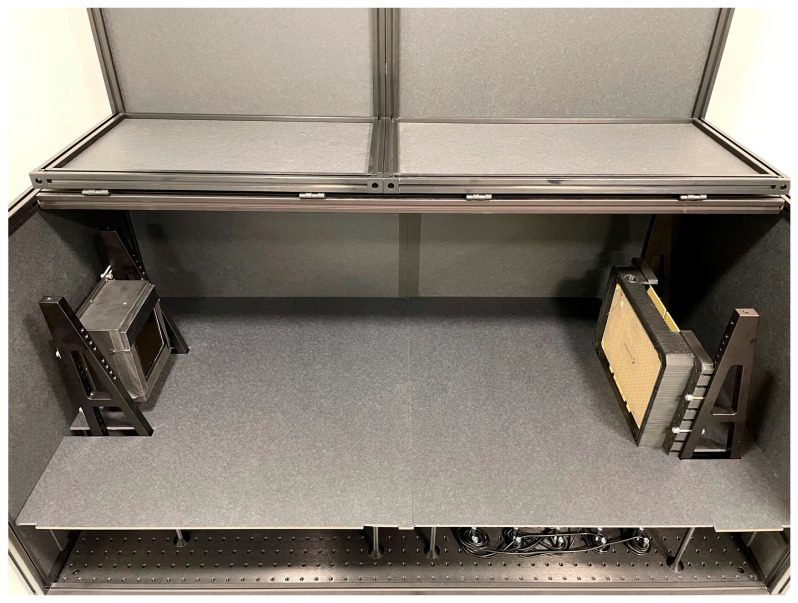
Dark box setup with the light source on the left and the sensor on the right. The sensor is on a black mechanical support.

The sensor was first characterised using the photon transfer curve method with visible light. The data was captured by placing the system within a dark box and sweeping the integration time with a constant intensity green LED light centred at 530 nm. Figure 13 shows the test setup used for characterisation with light, with the light source on the left and the sensor on the right. The optical testing was completed at room temperature and with no cooling or temperature control for the sensor. All the results shown in this section were obtained with the default bit depth of 9 bits.

**Figure 14:**
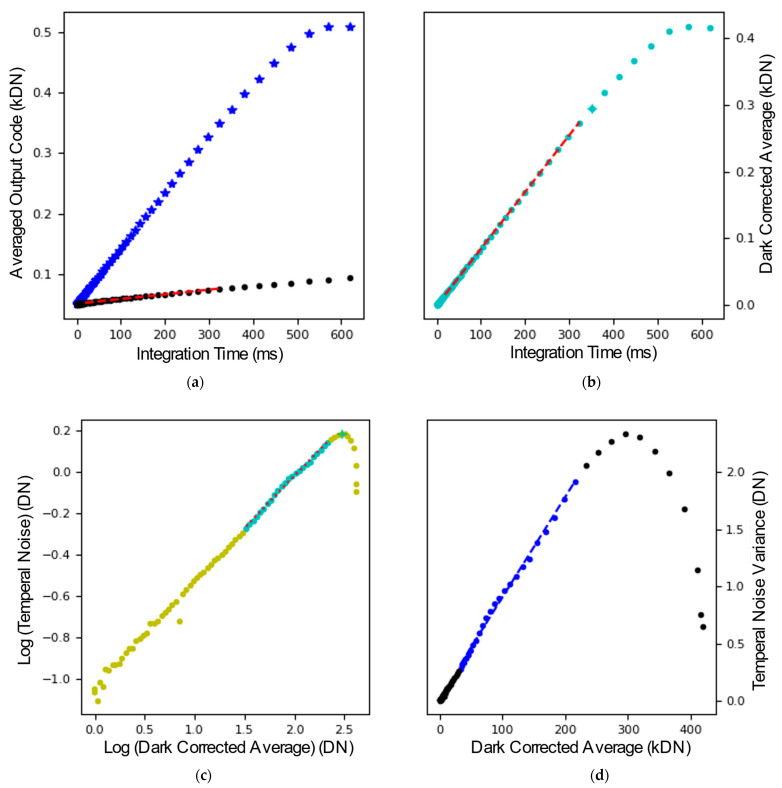
Characterisation of the sensor with visible light bypassing the PGA. (**a**) Transfer curve with the blue curve showing the sweep with light and the black curve showing the response without light. (**b**) Transfer curve subtracting the response in the dark from the response in the light shown in (**a**). (**c**) Control curve plotting the logarithm of the temporal noise versus the logarithm of the dark corrected average value. The light blue points show where the 0.5 slope was found and used to calculate the gain of the sensor. (**d**) shows the variance in the noise vs. the dark corrected average. The blue point shows the area from which the gain is being extracted.

**Figure 15:**
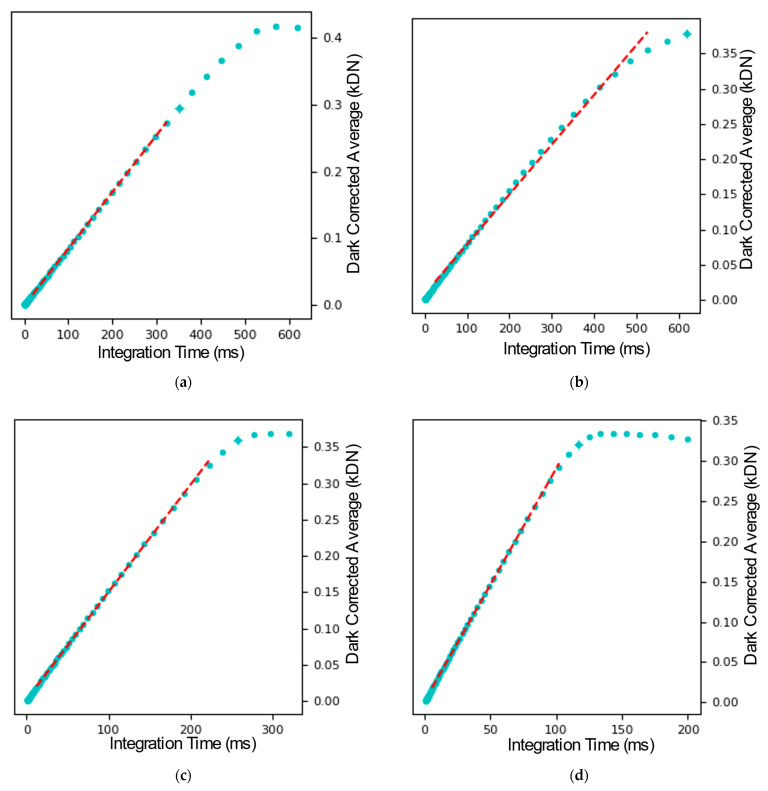
Transfer curves showing the dark subtracted sweep vs. integration time are shown for different PGA settings: (**a**) PGA Bypass, (**b**) PGA set with gain 1, (**c**) PGA set with gain 2, (**d**) PGA set with gain 4.

The transfer curves and photon-transfer curves are shown in Figure 14, and all parameters were extracted in accordance with the European Machine Vision Association (EMVA) 1288 Release 4 standard [22]. In order to extract the gain from the measurements, first, the region of the control curve with a 0.5 slope (Figure 14c) was determined. This region was identified in the photon transfer curve (Figure 14d) where a linear plot in this region gave the gain of the sensor. The linear full-well capacity was determined as the output for which the variance reaches its maximum, while the saturation full-well was identified at the point where the variance is equal to zero. The noise was calculated from the first point in the dark sweep, using the previously determined gain to convert the noise into units of electrons. The analysis was repeated with different configurations of the PGA with gains of 1×, 2×, and 4×. The measured transfer curves are shown in Figure 15. A summary of the parameters extracted from the optical test results is given in Table 1.

**Figure 16:**
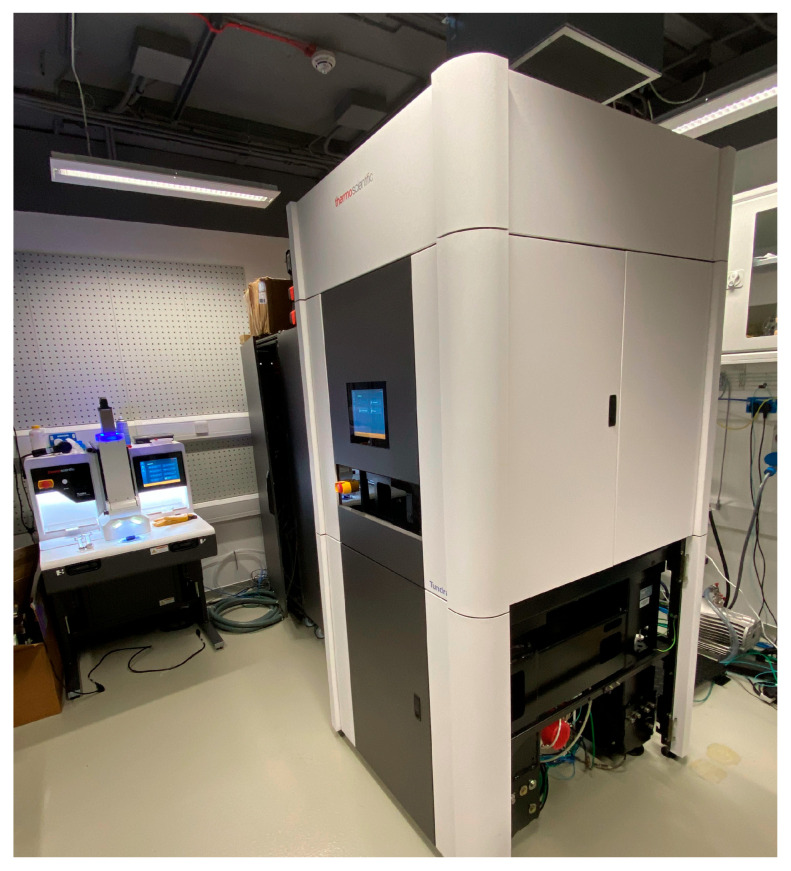
Picture of the tundra TEM microscope used for the testing of the sensor. Courtesy of Prof. Richard Henderson, MRC-LMB, Cambridge.

After the optical characterisation, the sensor was installed in a ThermoFisher Scientific Tundra Cryo-TEM microscope (Figure 16). The sensor sits within the column of the microscope, which is in a vacuum, and it is actively cooled and kept at a constant temperature that can be adjusted.

**Figure 17:**
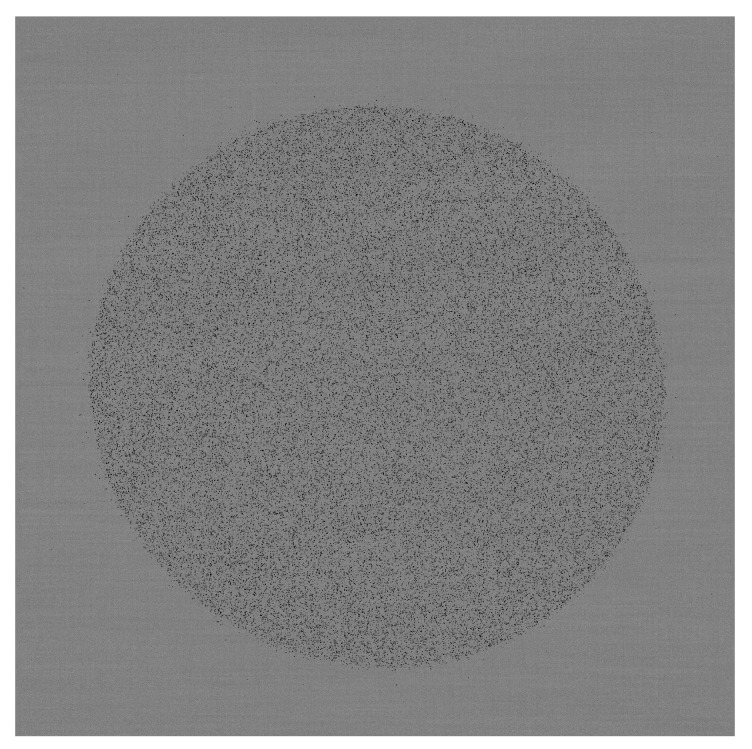
Single frame captured in a cryoEM, showing the image of a circular electron beam. In this frame and the following image, electron hits are shown as darker than the background. Courtesy of Dr Greg McMullan, MRC-LMB, Cambridge.

**Figure 18:**
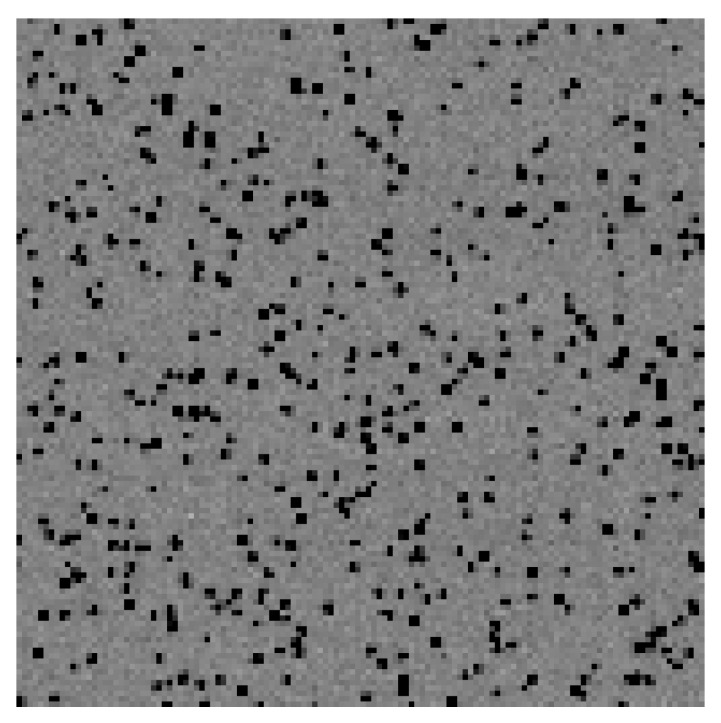
This figure shows a zoom to the central 128 × 128 pixels of the previous figure. Single electron hits are clearly visible. Courtesy of Dr Greg McMullan, MRC-LMB Cambridge.

**Figure 19:**
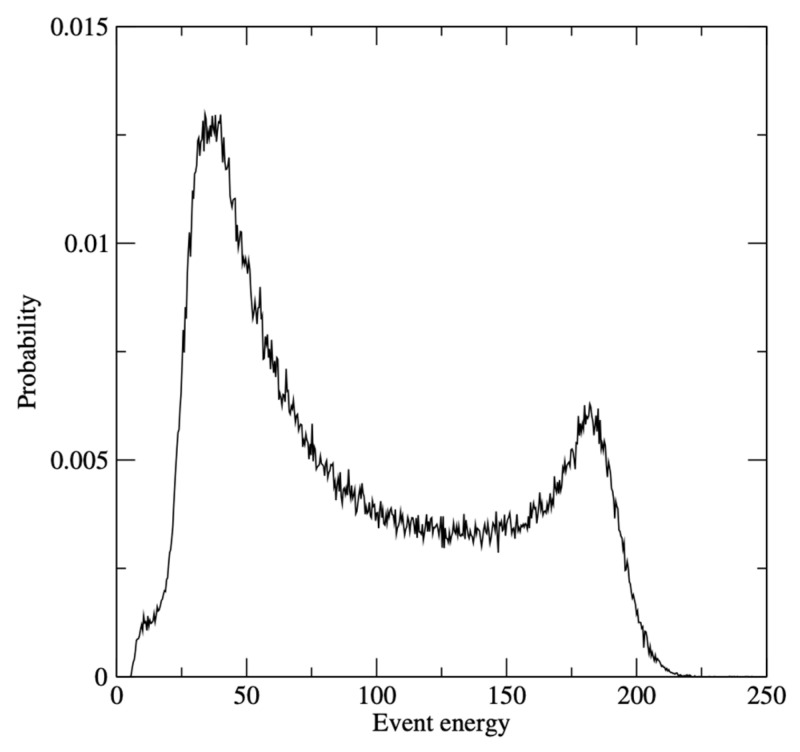
Energy loss distribution, i.e., measurement of the energy lost by every single electron. Courtesy of Dr Greg McMullan, MRC-LMB Cambridge.

A single frame recorded in this cryoEM setup is shown in Figure 17, where electron hits are shown as darker than the background. A zoom to the central 128 × 128 pixel area is shown in Figure 18. Single electron hits are clearly visible. It is also clear that the charge generated by a single electron can be shared between more than one pixel. By summing up the charge corresponding to a single electron hit, it is possible to reconstruct the total amount of charge generated by a single electron. A histogram of the distribution of generated charge can then be generated, corresponding to the so-called Landau curve [17]. The measured curve is shown in Figure 19. Two regions can be identified in this curve. The peak on the right corresponds to electrons that were fully absorbed in the silicon, thus releasing the whole 100 keV and generating a total of 27,777 e/h pairs. The rest of the curve corresponds to electrons that traversed the sensitive layer of the sensor without being fully absorbed and, as expected, has a peak on the left as well as a tail to the right for events with a higher released charge.

## 4. Conclusions

We have designed, manufactured, and characterised a high-speed wafer-scale CMOS image sensor. The design of the sensor has been explained in detail, showing that the implemented architectural choices allowed for meeting the demanding frame rate and bandwidth requirements of the sensor while balancing the practical constraints of the sensor’s implementation.

**Figure 20:**
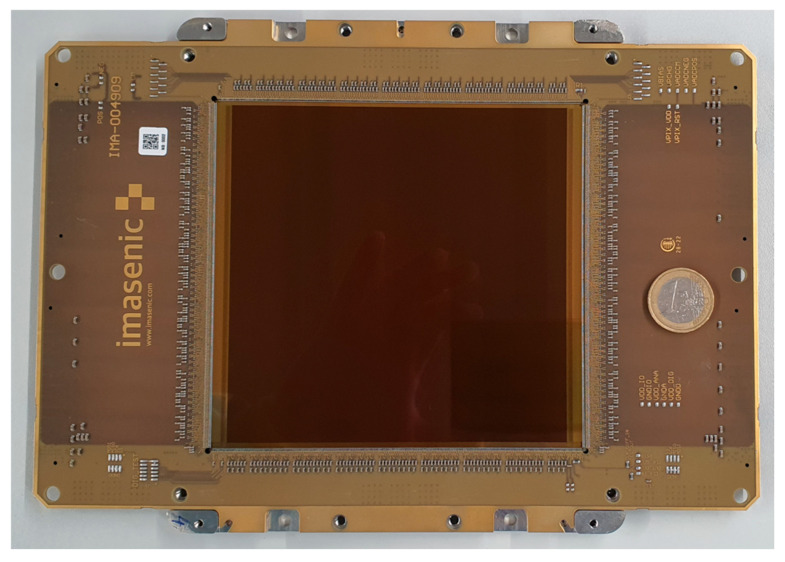
Photo of the sensor on its Chip-On-Board (COB). A 1-euro coin is also shown as a reference size.

A photo of the sensor is shown in Figure 20 and compared to the size of a 1-euro coin. The characteristics of the sensor are given in Table 2. Although only the 9-bit depth has been characterised, the sensor speed for the other bit depths has been derived from simulations based on the successful testing of the 9-bit mode. When read with 8-bit resolution, the sensor speed is in excess of 5000 fps with the full resolution of 4.2 Mpixels, thus achieving a pixel rate of 22.1 Gpixels per second. Until today, similar pixel rates were only achieved by conventional high-speed CIS, normally not larger than a reticle and much smaller than a full 200 mm CMOS wafer. The sensor was also successfully tested in a cryoEM environment, demonstrating that its performance meets expectations and enabling efficient detection of single electrons. The sensor is currently being used for high-resolution reconstruction of complex biological structures in a cryoEM.

## Figures and Tables

**Table 1:** Gain and Full Well Noise as measured with the PTC.

Parameter	Bypass PGA	PGA X1	PGA X2	PGA X4
Gain (DN/e−)	8.78	7.33	16.38	33.33
Linear Full Well (ke−)	33.67	51.61	21.93	9.63
Saturation Full Well (ke−)	47.65	51.6	22.54	9.63
Noise (e− rms)	86.4	107.5	68.2	55.2

**Table 2:** Parameters of the 4 Mpixel, high-speed, wafer-scale sensor.

Parameter	Value
Format	2052 × 2064 pixels 4.2 megapixels
Illuminated	Front Side Illuminated (FSI)
Pixel size	58 µm
Focal plane size	119.0 mm × 119.7 mm
Noise	86 e− rms
Linear full well	33,700 e−
ADC	Column-parallel
ADC resolution (User-selectable)	4, 6, 8, 9, and 10 bits
Frame rate (At full 4.2 Mpixels resolution)	7267 @ 4 bit 5768 @ 6 bits 5266 @ 8 bits 4542 @ 9 bits 3303 @ 10 bits
Frame rate (ROI: 2052 × 24)	303,000 @ 8 bits 189,394 @ 10 bits
Frame rate (ROI: 2052 × 1056)	6887 @ 8 bits 4304 @ 10 bits

## Data Availability

Data supporting the findings of this study are not publicly available due to confidentiality restrictions.

## Abbreviations

The following abbreviations are used in this manuscript:

PGA	Programmable Gain Amplifier
ADC	Analogue to Digital Converter
DDR	Double Data Rate
ROI	Region of Interest
CMOS	Complementary Metal Oxide Semiconductor
CIS	CMOS Image Sensor
COB	Chip-On-Board
FPN	Fixed-Pattern Noise
SPI	Serial-Parallel Interface
PPPD	Partially Pinned Photodiode
HS	Half Select
LVDS	Low-Voltage Differential Signalling
CryoEM	Cryogenic Electron Microscopy
TEM	Transmission Electron Microscope
S/H	Sample and Hold
FSI	Front Side Illuminated

## References

[1] Farrier M.G., Achterkirchen T.G., Weckler G.P., Bosiers J. CMOS Active Pixel Detectors for Radiography. Proceedings of the 2009 Image Sensor Workshop.

[2] Assaf L., Tomer L., Raz R., Amos F. Enhanced X-RAY CMOS Sensor Panel for Radio and Fluoro Application Using a Low Noise Charge Amplifier Pixel with a Partially Pinned PD. Proceedings of the 2011 International Image Sensor Workshop.

[3] Korthout L., Verbugt D., Timpert J., Mierop A., de Haan W., Maes W., de Meulmeester J., Muhammad W., Dillen B., Stoldt H. A Wafer-Scale CMOS APS Imager for Medical X-Ray Applications. Proceedings of the 2009 International Image Sensor Workshop.

[4] Sedgwick I., Das D., Guerrini N., Marsh B., Turchetta R. LASSENA: A 6.7 Megapixel, 3-Sides Buttable Wafer-Scale CMOS Sensor Using a Novel Grid- Addressing Architecture. Proceedings of the 2013 International Image Sensor Workshop.

[5] Van Blerkom D., Truong L., Rysinski J., Corlan R., Venkatesan K., Bagwell S., Oniciuc L., Bergey J. A 1Mpixel, 80k Fps Global Shutter CMOS Image Sensor for High Speed Imaging. Proceedings of the 2021 International Image Sensor Workshop.

[6] Blanchaert T., Ceulemans B., Wolfs B., Cotteleer W., Lepage G., Vanhorebeek G., Markey E., Huysman A., Jiang T., Li Y. High Speed 21M Pixel Global Shutter Image Sensor. Proceedings of the 2021 International Image Sensor Workshop.

[7] Agarwal A., Hansrani J., Bagwell S., Rytov O., Shah V., Ong K.L., Blerkom D.V., Bergey J., Kumar N., Lu T. (2023). A 316MP, 120FPS, High Dynamic Range CMOS Image Sensor for Next Generation Immersive Displays. Sensors.

[8] Kühlbrandt W. (2014). The Resolution Revolution. Science.

[9] RCSB PDB PDB Statistics: Number of Released PDB Structures per Year. https://www.rcsb.org/stats/all-released-structures.

[10] Lavery L. Cryo-EM Used in Novel Coronavirus Research to Support Vaccine, Treatment Development. https://www.thermofisher.com/blog/atomic-resolution/cryo-em-used-in-novel-coronavirus-research-to-support-vaccine-treatment-development/.

[11] Lynch M.L., Snell E.H., Bowman S.E.J. (2021). Structural Biology in the Time of COVID-19: Perspectives on Methods and Milestones. IUCrJ.

[12] Faruqi A.R., Henderson R., McMullan G. (2015). Progress and Development of Direct Detectors for Electron Cryomicroscopy. Advances in Imaging and Electron Physics.

[13] Peet M.J., Henderson R., Russo C.J. (2019). The Energy Dependence of Contrast and Damage in Electron Cryomicroscopy of Biological Molecules. Ultramicroscopy.

[14] Chan L.M., Courteau B.J., Maker A., Wu M., Basanta B., Mehmood H., Bulkley D., Joyce D., Lee B.C., Mick S. (2024). High-Resolution Single-Particle Imaging at 100–200 keV with the Gatan Alpine Direct Electron Detector. J. Struct. Biol..

[15] Karia D., Koh A.F., Yang W., Cushing V.I., Basanta B., Mihaylov D.B., Khavnekar S., Vyroubal O., Malínský M., Sháněl O. (2025). Sub-3 Å Resolution Protein Structure Determination by Single-Particle Cryo-EM at 100 keV. Structure.

[16] Durini D. (2014). High Performance Silicon Imaging: Fundamentals and Applications of CMOS and CCD Sensors.

[17] Bichsel H. (1988). Straggling in Thin Silicon Detectors. Rev. Mod. Phys..

[18] Dectris DECTRIS SINGLA Web Brochure. https://media.dectris.com/filer_public/25/87/25871486-5561-4af1-8ba5-6f28e9fa6871/singla_web_brochure_dectris_15_01.pdf.

[19] Hutchings S.W., Larsen H., Sharkawy M.E., Sedgwick I., Marsh B., Starborg T., Barnard J., Hart M., Macwaters C., Vassilev D. C100: A 4-Megapixel, 2000 FPS Wafer Scale Direct Electron Detection Sensor for 100keV Electron Cyro-Microscopy. Proceedings of the C100: A 4-Megapixel, 2000 FPS Wafer Scale Direct Electron Detection Sensor for 100keV Electron Cyro-Microscopy.

[20] Sannino M., Bofill-Petit A., Giulioni M., Mollà Garcia A., Turchetta R., McMullan G., Henderson R., Copetti C., Janssen B., Mele L. A Rad-Hard, 60µm Pixel Sensor Optimized for the Direct Detection of Electrons. Proceedings of the 2021 International Image Sensor Workshop.

[21] Saks N.S., Ancona M.G., Modolo J.A. (1984). Radiation Effects in MOS Capacitors with Very Thin Oxides at 80°K. IEEE Trans. Nucl. Sci..

[22] Sannino M., Bofill-Petit A., Pinaroli G., Turchetta R. A High Dynamic Range, 1.9 Mpixel CMOS Image Sensor for X-Ray Imaging with In-Pixel Charge Binning and Column Parallel ADC. Proceedings of the 2019 International Image Sensor Workshop.

